# Correction to: NHR-23 and SPE-44 regulate distinct sets of genes during *Caenorhabditis elegans* spermatogenesis

**DOI:** 10.1093/g3journal/jkad048

**Published:** 2023-03-07

This is a correction to: James Matthew Ragle, Kayleigh N Morrison, An A Vo, Zoe E Johnson, Javier Hernandez Lopez, Andreas Rechtsteiner, Diane C Shakes, Jordan D Ward, NHR-23 and SPE-44 regulate distinct sets of genes during *Caenorhabditis elegans* spermatogenesis, *G3 Genes*|*Genomes*|*Genetics*, Volume 12, Issue 11, November 2022, jkac256, https://doi.org/10.1093/g3journal/jkac256

In the originally published online version of this manuscript, in Table 2 all nsph genes were incorrectly listed as MFP1 paralogs instead of as MFP2 paralogs.

This error has now been corrected.

