# Supplementary material for: NHR-23 and SPE-44 regulate distinct sets of genes during Caenorhabditis elegans spermatogenesis
Source: G3 (Bethesda). 2022 Sep 22;12(11):jkac256. doi: 10.1093/g3journal/jkac256 (PMC9635660; doi:10.1093/g3journal/jkac256)
Supplement: jkac256_Supplementary_Figure_S1 [file jkac256_supplementary_figure_s1.pdf]

A)

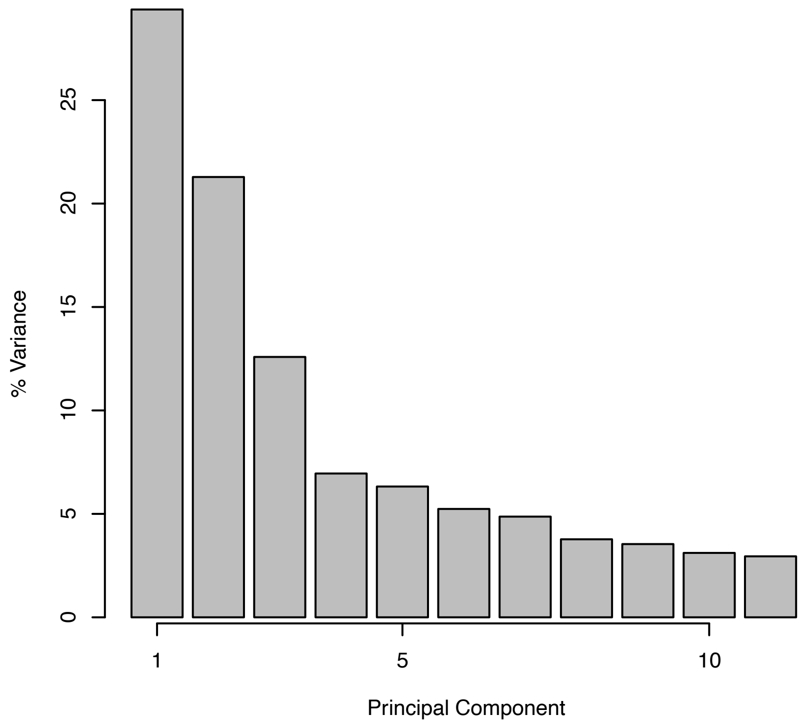


B)

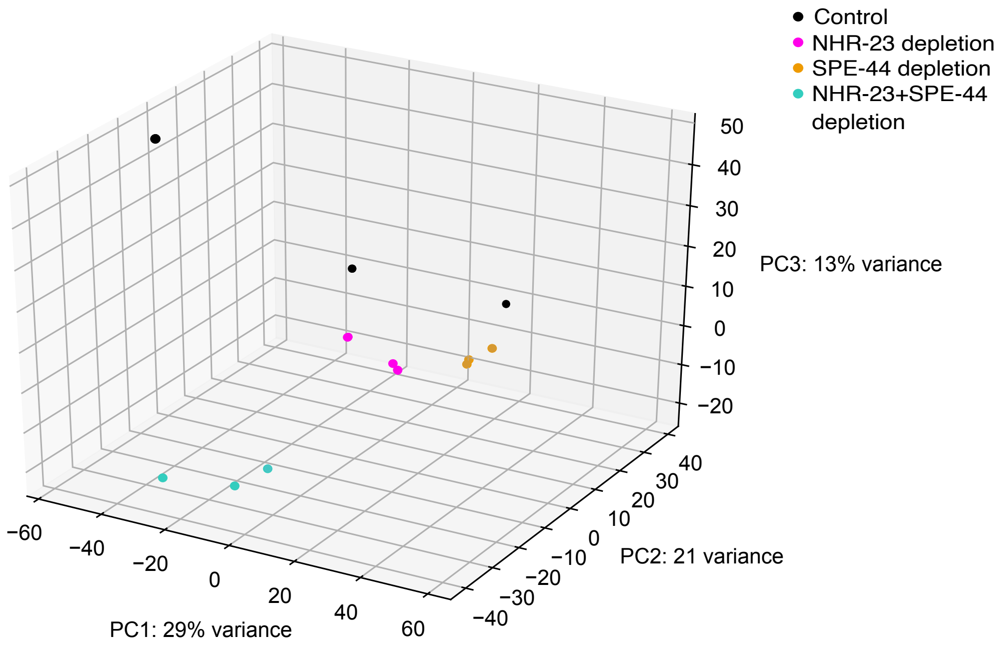


**Figure S1. Analysis of RNA-seq dataset variance.** (A) Scree plot depicting the percent variance associated with eleven principal components. (B) 3D Principal component analysis of RNA-seq data. Legend indicates which colored dots belong to control, NHR-23-depleted, SPE-44-depleted, or NHR-23+SPE-44-depleted biological replicates.
