# Supplementary material for: NHR-23 and SPE-44 regulate distinct sets of genes during Caenorhabditis elegans spermatogenesis
Source: G3 (Bethesda). 2022 Sep 22;12(11):jkac256. doi: 10.1093/g3journal/jkac256 (PMC9635660; doi:10.1093/g3journal/jkac256)
Supplement: jkac256_Supplementary_Figure_S2 [file jkac256_supplementary_figure_s2.pdf]

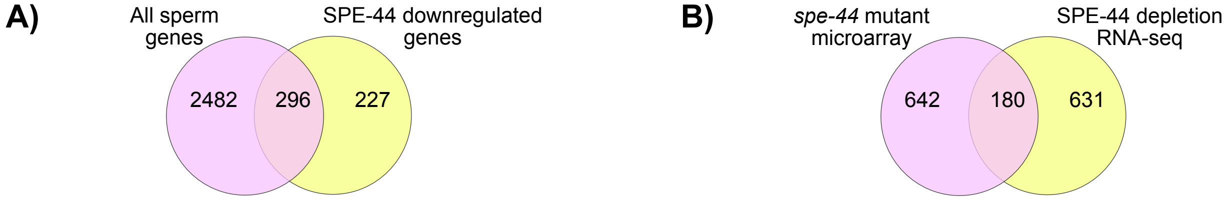

Figure S2. Overlaps between SPE-44-downregulated genes and sperm-enriched genes, and comparison of SPE-44 RNA-seq and *spe-44* microarray datasets. (A) Venn diagram of down-regulated genes following SPE-44 depletion and sperm-enriched genes. (B)Venn diagram of genes differentially regulated following SPE-44 depletion (this study) and a previous microarray experiment comparing gene expression in L4 males between *spe-44* null mutants and wild-type animals (Kulkarni et al. 2012).
