# Supplementary material for: NHR-23 and SPE-44 regulate distinct sets of genes during Caenorhabditis elegans spermatogenesis
Source: G3 (Bethesda). 2022 Sep 22;12(11):jkac256. doi: 10.1093/g3journal/jkac256 (PMC9635660; doi:10.1093/g3journal/jkac256)
Supplement: jkac256_Supplementary_Figure_S3 [file jkac256_supplementary_figure_s3.pdf]

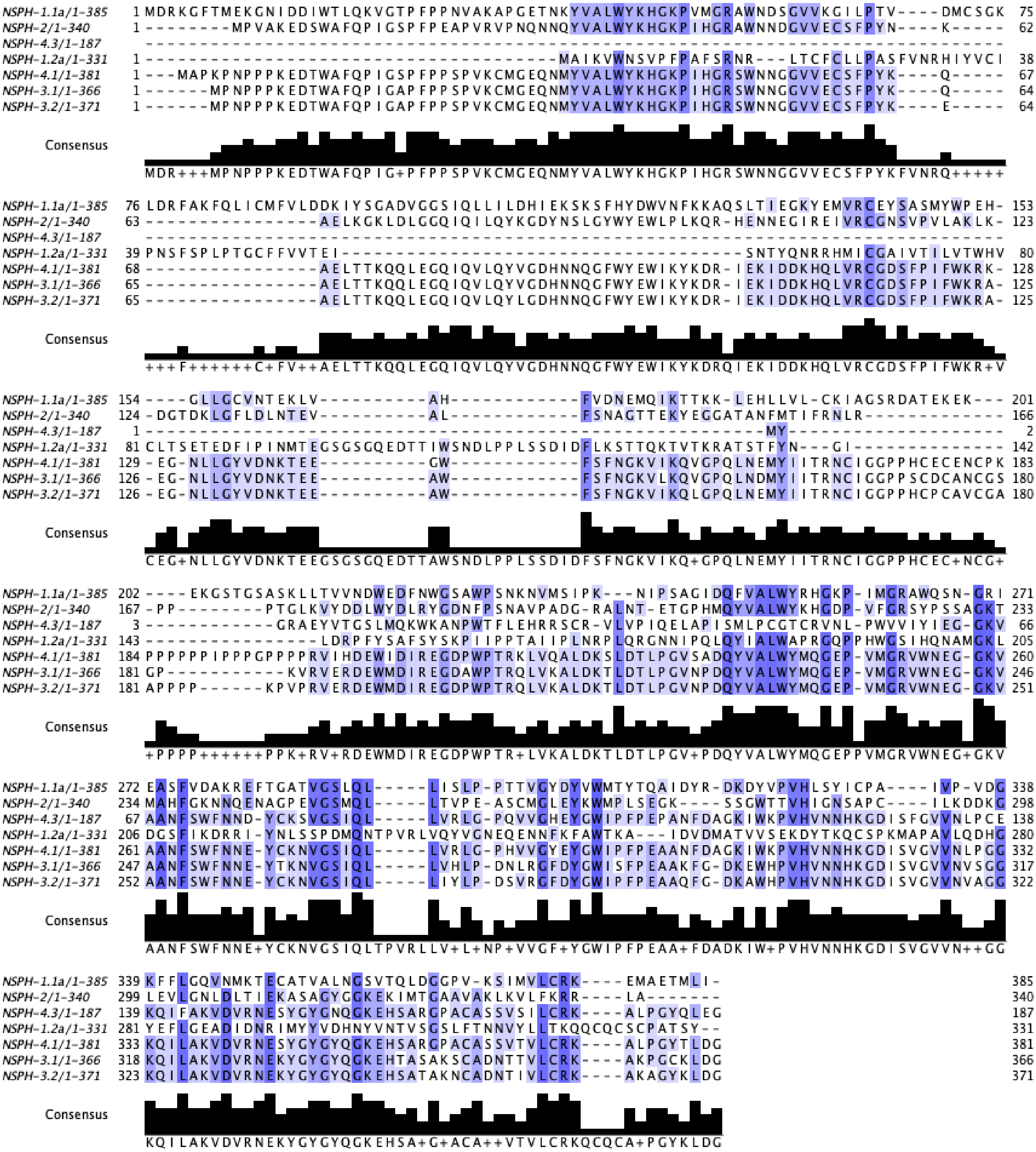

Figure S3. NSPH paralog alignment. An alignment produced with Clustal Omega (Goujon et al., 2010; Sievers et al., 2011) and visualized with JalView2 (Waterhouse et al., 2009). Protein identity for each row and total length in amino acids is provided in the left column. To the left and right of the alignment are amino acid position references. The consensus sequence is shown beneath each row. Only the sequence for NSPH-4.1 is shown as NSPH-4.2 has identical sequence and its protein product is annotated as NSPH-4.1 in Wormbase.
