# Supplementary material for: NHR-23 and SPE-44 regulate distinct sets of genes during Caenorhabditis elegans spermatogenesis
Source: G3 (Bethesda). 2022 Sep 22;12(11):jkac256. doi: 10.1093/g3journal/jkac256 (PMC9635660; doi:10.1093/g3journal/jkac256)
Supplement: jkac256_Supplementary_Figure_S4 [file jkac256_supplementary_figure_s4.pdf]

Figure S4. MFP2 genomic DNA alignment. An alignment produced with Clustal Omega (Goujon et al., 2010; Sievers et al., 2011). cDNA identity for each row and total length in basepairs is provided in the right column. An alternate potential *ZK596.2* reading frame starting 92 bp downstream from the conserved start codon is shown in *ZK596.2alt*.

```
nsph-4.3        ------------------------------------------------------------ 0
nsph-4.1        ATGGCGCCGAAGCCGAATCCGCCACCAAAAGAAGACACAT-GGGCTTTCCAGCCGATTGG 59
nsph-4.2        ATGGCGCCGAAGCCGAATCCGCCACCAAAAGAAGACACAT-GGGCTTTCCAGCCGATTGG 59
ZK546.7         ATGACGCCGAAGCCGAATCCGCCACCAAAAGAAGACACATGGAGCGTTCCAGCCTATTGG 60
ZK546.7alt      ------------------------------------------------------------ 0

nsph-4.3        ------------------------------------------------------------ 0
nsph-4.1        TTCACCGTTCCCGCCGAGTCCTGTCAAATGTATGGGAGAGCAGAACATGTATGTTGCTCT 119
nsph-4.2        TTCACCGTTCCCGCCGAGTCCTGTCAAATGTATGGGAGAGCAGAACATGTATGTTGCTCT 119
ZK546.7         ATCCCCATTTCCGCCGAGCCCCGTCAAGTGTATGGGAGAGCAGAACATGTATGTGGCTCT 120
ZK546.7alt      -------------------------------ATGGGAGAGCAGAACATGTATGTGGCTCT 29

nsph-4.3        ------------------------------------------------------------ 0
nsph-4.1        CTGGTACAAACATGGTAAACCAATTCATGGACGTTCCTGGAACAACGGAGGAGTGGTTGA 179
nsph-4.2        CTGGTACAAACATGGTAAACCAATTCATGGACGTTCCTGGAACAACGGAGGAGTGGTTGA 179
ZK546.7         CTGGTACAAACATGGCAAGCCAATTCATGGGCGTTCCTGGAATAACGGAGGAGTCGTTGA 180
ZK546.7alt      CTGGTACAAACATGGCAAGCCAATTCATGGGCGTTCCTGGAATAACGGAGGAGTCGTTGA 89

nsph-4.3        ------------------------------------------------------------ 0
nsph-4.1        ATGCTCATTCCCATACAAGCAGGCAGAGTTGACGACCAAGCAACAGCTCGAGGGACAAAT 239
nsph-4.2        ATGCTCATTCCCATACAAGCAGGCAGAGTTGACGACCAAGCAACAGCTCGAGGGACAAAT 239
ZK546.7         ATGCTCCTTCCCATACAAGGAGGCAGAGTTGACGACCAAGCAACAGCTCGAAGGACAAAT 240
ZK546.7alt      ATGCTCCTTCCCATACAAGGAGGCAGAGTTGACGACCAAGCAACAGCTCGAAGGACAAAT 149

nsph-4.3        ------------------------------------------------------------ 0
nsph-4.1        TCAGGTGCTCCAGTATGTCGGAGATCACAACAACCAAGGATTCTGGTATGAATGGATCAA 299
nsph-4.2        TCAGGTGCTCCAGTATGTCGGAGATCACAACAACCAAGGATTCTGGTATGAATGGATCAA 299
ZK546.7         CCAGGTTCTGCAGTATGTTGGAGATCACAACAACCAAGGATTCTGGTATGAATGGATCAA 300
ZK546.7alt      CCAGGTTCTGCAGTATGTTGGAGATCACAACAACCAAGGATTCTGGTATGAATGGATCAA 209

nsph-4.3        ------------------------------------------------------------ 0
nsph-4.1        ATACAAGGATCGTATTGAGAAGATCGACGACAAACATCAACTTGTTCGCTGTGGAGATTC 359
nsph-4.2        ATACAAGGATCGTATTGAGAAGATCGACGACAAACATCAACTTGTTCGCTGTGGAGATTC 359
ZK546.7         ATACAAGGATCGCATTGAGAAGATCGATGACAAGCATCAACTTGTTCGTTGTGGGGATTC 360
ZK546.7alt      ATACAAGGATCGCATTGAGAAGATCGATGACAAGCATCAACTTGTTCGTTGTGGGGATTC 269

nsph-4.3        ------------------------------------------------------------ 0
nsph-4.1        GTTCCCAATCTTCTGGAAGAGAAAGGAAGGCAATCTTCTTGGATACGTTGACAATAAGAC 419
nsph-4.2        GTTCCCAATCTTCTGGAAGAGAAAGGAAGGCAATCTTCTTGGATACGTTGACAATAAGAC 419
ZK546.7         TTTCCCAATCTTCTGGAAGAGAAAGGAAGGAAATCTCCTTGGATACGTCGACAACAAGAC 420
ZK546.7alt      TTTCCCAATCTTCTGGAAGAGAAAGGAAGGAAATCTCCTTGGATACGTCGACAACAAGAC 329

nsph-4.3        ------------------------------------------------------------ 0
nsph-4.1        GGAGGAAGGATGGTTCTCGTTCAATGGCAAGGTGATCAAACAAGTCGGTCCACAGTTGAA 479
nsph-4.2        GGAGGAAGGATGGTTCTCGTTCAATGGCAAGGTGATCAAACAAGTCGGTCCACAGTTGAA 479
ZK546.7         AGAGGAAGGATGGTTCTCGTTCAACGGAAAAGTGATCAAACAAGTCGGACCACAGTTGAA 480
ZK546.7alt      AGAGGAAGGATGGTTCTCGTTCAACGGAAAAGTGATCAAACAAGTCGGACCACAGTTGAA 389
```

```
nsph-4.3        ------------------------------------------------------------ 0
nsph-4.1        TGAAATGTACATCATCACTCGCAACTGCATCGGAGGACCACCACACTGCGAATGCGAGAA 539
nsph-4.2        TGAAATGTACATCATCACTCGCAACTGCATCGGAGGACCACCACACTGCGAATGCGAGAA 539
ZK546.7         TGACATGTACATCATCACTCGTAACTGCGTCGGAGGACCACCATACTGCGAGTGTGAGAA 540
ZK546.7alt      TGACATGTACATCATCACTCGTAACTGCGTCGGAGGACCACCATACTGCGAGTGTGAGAA 449

nsph-4.3        -------------------------------------ATGTATGGGAGAGCAGAATATG- 22
nsph-4.1        CTGTCCTAAACCACCACCACCACCACCAATCCCACCACCGGGCCCACCACCACCACGTGT 599
nsph-4.2        CTGTCCTAAACCACCACCACCACCACCAATCCCACCACCGGGCCCACCACCACCACGTGT 599
ZK546.7         CTGTCCGAAGCTTCCGCCACCACCACCAATCCCACCACCGGGCCCACCACCACCACGTGT 600
ZK546.7alt      CTGTCCGAAGCTTCCGCCACCACCACCAATCCCACCACCGGGCCCACCACCACCACGTGT 509
                                                       *        * **  *  **

nsph-4.3        -T-AACTGGCTCTCTGATGCAAAAATGGAAAGCCAATCCATGGACGTTCCTGGAA----C 76
nsph-4.1        CATACACGACGAATGGATCGATATCCGAGAGGGAGATCCATGGCCTACTCGCAAGCTTGT 659
nsph-4.2        CATACACGACGAATGGATCGATATCCGAGAGGGAGATCCATGGCCTACTCGCAAGCTTGT 659
ZK546.7         CATGCACGACGAATGGATCGACATCCGCGAGGGAGATCCATGGCCAACTCGCAAGCTTGT 660
ZK546.7alt      CATGCACGACGAATGGATCGACATCCGCGAGGGAGATCCATGGCCAACTCGCAAGCTTGT 569
                       * *     ***  * *   *  * *   ******** *    *   *

nsph-4.3        ACCGGAGGAGTTGTCGAGTGCTCGTTCCCATACAGGAGTTAGCGCCGATCAGTATGTTGC 136
nsph-4.1        TCAAGCTTTGGATAAGTCTTTGGATACTCTTCCAGGAGTCAGTGCCGATCAATATGTTGC 719
nsph-4.2        TCAAGCTTTGGATAAGTCTTTGGATACTCTTCCAGGAGTCAGTGCCGATCAATATGTTGC 719
ZK546.7         TAAAGCTTTGGACAAATCCTTGGACACCCTTCCAGGAGTCAGTGCAGATCAGTATGTTGC 720
ZK546.7alt      TAAAGCTTTGGACAAATCCTTGGACACCCTTCCAGGAGTCAGTGCAGATCAGTATGTTGC 629
                    *    *                * * * ******* ** ** ***** ********

nsph-4.3        CTTGTGGTACATGCAGGGTGAACCTGCCATGGGTCGTGATTTATATCGAGGGAGGAAAGG 196
nsph-4.1        CTTGTGGTACATGCAGGGTGAACCTGTTATGGGTCGTGTCTGGA-ATGAGGGAGGAAAGG 778
nsph-4.2        CTTGTGGTACATGCAGGGTGAACCTGTTATGGGTCGTGTCTGGA-ATGAGGGAGGAAAGG 778
ZK546.7         CTTGTGGTACATGCAGGGTGAACCTGTCATGGGTCGTGTTTGGA-ATGAGGGAGGAAAGG 779
ZK546.7alt      CTTGTGGTACATGCAGGGTGAACCTGTCATGGGTCGTGTTTGGA-ATGAGGGAGGAAAGG 688
                **************************  **********  *      *************

nsph-4.3        TTGCAGCCAACTTCTCTTGGTTCAACAACGATTACTGCAAGAGCGTTGGTTCCATTCAGT 256
nsph-4.1        TTGCTGCCAACTTCTCTTGGTTCAACAACGAATACTGCAAGAACGTTGGATCCATTCAAT 838
nsph-4.2        TTGCTGCCAACTTCTCTTGGTTCAACAACGAATACTGCAAGAACGTTGGATCCATTCAAT 838
ZK546.7         TTGCCGCCAACTTCTCTTGGTTCAACAATGAATATTGCAAGAACGTTGGCTCCATTCAGT 839
ZK546.7alt      TTGCCGCCAACTTCTCTTGGTTCAACAATGAATATTGCAAGAACGTTGGCTCCATTCAGT 748
                **** *********************** ** ** ******* ****** ******** *

nsph-4.3        TGCTTGTACGTCTCGGACCACAGGTTGTTGGACACGAGTATGGATGGATTCCATTCCCAG 316
nsph-4.1        TGCTTGTTCGTCTTGGACCACACGTCGTTGGATACGAGTATGGATGGATTCCATTCCCAG 898
nsph-4.2        TGCTTGTTCGTCTTGGACCACACGTCGTTGGATACGAGTATGGATGGATTCCATTCCCAG 898
ZK546.7         TGCTCGTCCGTCTCGGACCACACGTCGTTGGATACGAATATGGTTGGATTCCATTCCCAG 899
ZK546.7alt      TGCTCGTCCGTCTCGGACCACACGTCGTTGGATACGAATATGGTTGGATTCCATTCCCAG 808
                **** ** ***** ******** ** ****** **** ***** ****************

nsph-4.3        AACCTGCCAACTTCGATGCTGGAAAAATCTGGAAGCCAGTGCACGTTAACAATCACAAGG 376
nsph-4.1        AAGCTGCCAACTTTGATGCTGGAAAGATCTGGAAGCCAGTTCACGTTAACAACCACAAGG 958
nsph-4.2        AAGCTGCCAACTTTGATGCTGGAAAGATCTGGAAGCCAGTTCACGTTAACAACCACAAGG 958
ZK546.7         AAGCTGCAAACTTCGACGCTGGAAAGATCTGGAAGCCAGTGCACGTCAACAACCACAAGG 959
ZK546.7alt      AAGCTGCAAACTTCGACGCTGGAAAGATCTGGAAGCCAGTGCACGTCAACAACCACAAGG 868
                ** **** ***** ** ******** ************** ***** ***** *******

nsph-4.3        GAGACATCTCTTTTGGAGTCGTTAACCTCCCATGTGAAAAGCAAATCTTTGCCAAAGTCG 436
nsph-4.1        GAGACATCTCAGTTGGAGTCGTTAACCTGCCAGGCGGAAAACAGATCCTTGCCAAGGTTG 1018
nsph-4.2        GAGACATCTCAGTTGGAGTCGTTAACCTTCCAGGCGGAAAACAGATCCTTGCCAAGGTTG 1018
ZK546.7         GAGATATTTCTGTTGGAGTGGTTAACCTTCCAGGCGGAAAGCAGATCCTTGCCAAGGTCG 1019
ZK546.7alt      GAGATATTTCTGTTGGAGTGGTTAACCTTCCAGGCGGAAAGCAGATCCTTGCCAAGGTCG 928
                **** ** **  ******* ******** *** * * *** ** *** ******* ** *
```

```
nsph-4.3        ATGTTCGTAACGAGTCTTATGGATATGGTAACCAAGGAAAGGAACACTCGGCTCGTGGAC 496
nsph-4.1        ATGTTCGCAACGAGTCGTACGGATATGGTTACCAAGGAAAGGAACACTCGGCTCGTGGAC 1078
nsph-4.2        ATGTTCGCAACGAGTCGTACGGATATGGTTACCAAGGAAAGGAACACTCAGCTCGTGGAC 1078
ZK546.7         ATGTTCGCAACGAGTCGTACGGATACGGTTATCAAGGAAAGGAGCACTCGGCTCGTGGAC 1079
ZK546.7alt      ATGTTCGCAACGAGTCGTACGGATACGGTTATCAAGGAAAGGAGCACTCGGCTCGTGGAC 988
                ******* ******** ** ***** *** * *********** ***** **********

nsph-4.3        CAGCTTGTGCATCCAGCGTCAGTATTCTTTGCCGCAAGGCTTTGCCAGGATACCAACTCG 556
nsph-4.1        CAGCTTGTGCCTCCAGTGTCACAGTTCTTTGCCGCAAGGCATTGCCAGGATACACCCTCG 1138
nsph-4.2        CAGCTTGTGCCTCCAGTGTCACAGTTCTTTGCCGCAAGGCATTGCCAGGATACACCCTCG 1138
ZK546.7         CTGCTTGTGCTTCGAGTGTCACTGTTCTTTGCCGCAAGGCTCTCCCAGGATACCAACTCG 1139
ZK546.7alt      CTGCTTGTGCTTCGAGTGTCACTGTTCTTTGCCGCAAGGCTCTCCCAGGATACCAACTCG 1048
                * ******** ** ** ****   ****************  * *********   ****

nsph-4.3        AGGGATAG  564
nsph-4.1        ACGGATAG  1146
nsph-4.2        ACGGATAG  1146
ZK546.7         ACGGATAG  1147
ZK546.7alt      ACGGATAG  1056
                * ******
```

Figure S4
